# Supplementary material for: Structural insights into the interaction between adenovirus C5 hexon and human lactoferrin
Source: J Virol. 2024 Feb 7;98(3):e01576-23. doi: 10.1128/jvi.01576-23 (PMC10949841; doi:10.1128/jvi.01576-23)
Supplement: Supplemental material — Figures S1 to S8; Table S1. [file jvi.01576-23-s0001.docx]

**
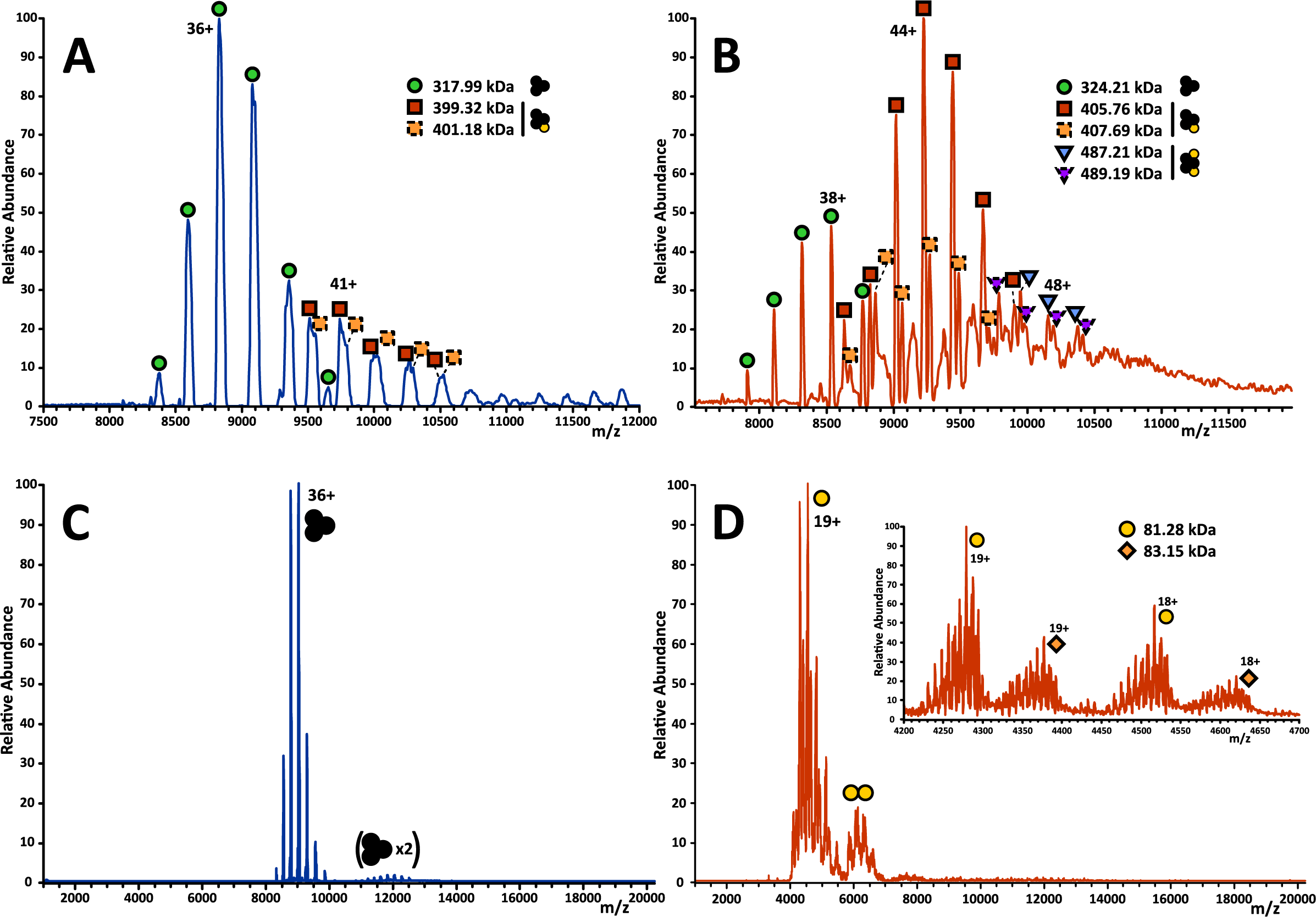
**

**Fig. S1.** Detailed native MS peak annotation. Close-up at higher (3.000) instrument resolving power setting shows details of detected 0.5 µM hexon:hLF complex species in 20 mM (A) and 5.85 mM (B) ammonium acetate pH 7.0. While hexon alone (C) is trimeric and highly homogeneous (5 µM in 150 mM ammonium acetate pH 7.0), hLF alone (D - at 5 µM in 150 mM AA pH 7.0) shows major heterogeneity at 12.000 MS resolving power setting stemming from the presence of two major proteoforms differing by 1.86 kDa as well as a broad distribution of hexose units (inset). This in turn explains the broad multi-apex peaks of hLF-containing complexes detected in A and B.

**Table S1.** Cryo-EM data collection, and model quality parameters

|  | **HAdV-C5 hexon**  (EMD- 18212)  (PDB- 8Q7C) |
| --- | --- |
| **Data collection and processing** |  |
| Microscope | Titan Krios |
| Detector | K2 LS |
| Magnification (nominal) | 165.000x |
| Voltage (kV) | 300 |
| Spherical abberation | 2.7 mm |
| Total electron dose (e^-^/Å^2^) | 55 |
| Defocus range (μm) | -2.5 to 1.2 |
| Pixel size (Å) | 0.82 |
| Number of Micrographs | 1063 |
| Final particle images (no.) | 53396 |
| Map resolution (Å)  FSC threshold | 2.96 (FSC_0.143_) |
| **Refinement** |  |
| Initial model used (PDB code) | 3TG7 |
| RMSZ  Bond lengths  Bond angles | 0.31  0.50 |
| Validation  MolProbity score  Clashscore, all-atom  Poor rotamers | 2.03  7.61  3.7% |
| Ramachandran plot  Favored  Allowed  Outliers | 96.92%  3.08%  0% |


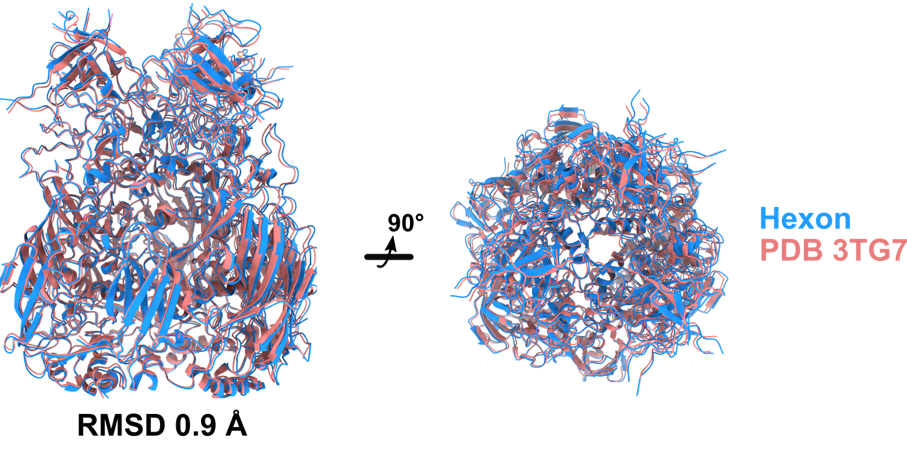


**Fig. S2.** Comparison of the HAdV-C5 hexon model obtained by single-particle cryoEM with a crystal structure of the same protein. The hexon model built using the cryo-EM reconstruction is depicted in blue (PDB: 8Q7C), the crystal structure (PDB: 3TG7) is shown in pink. Both structures are almost identical and can be aligned to an RMSD of 0.9Å.


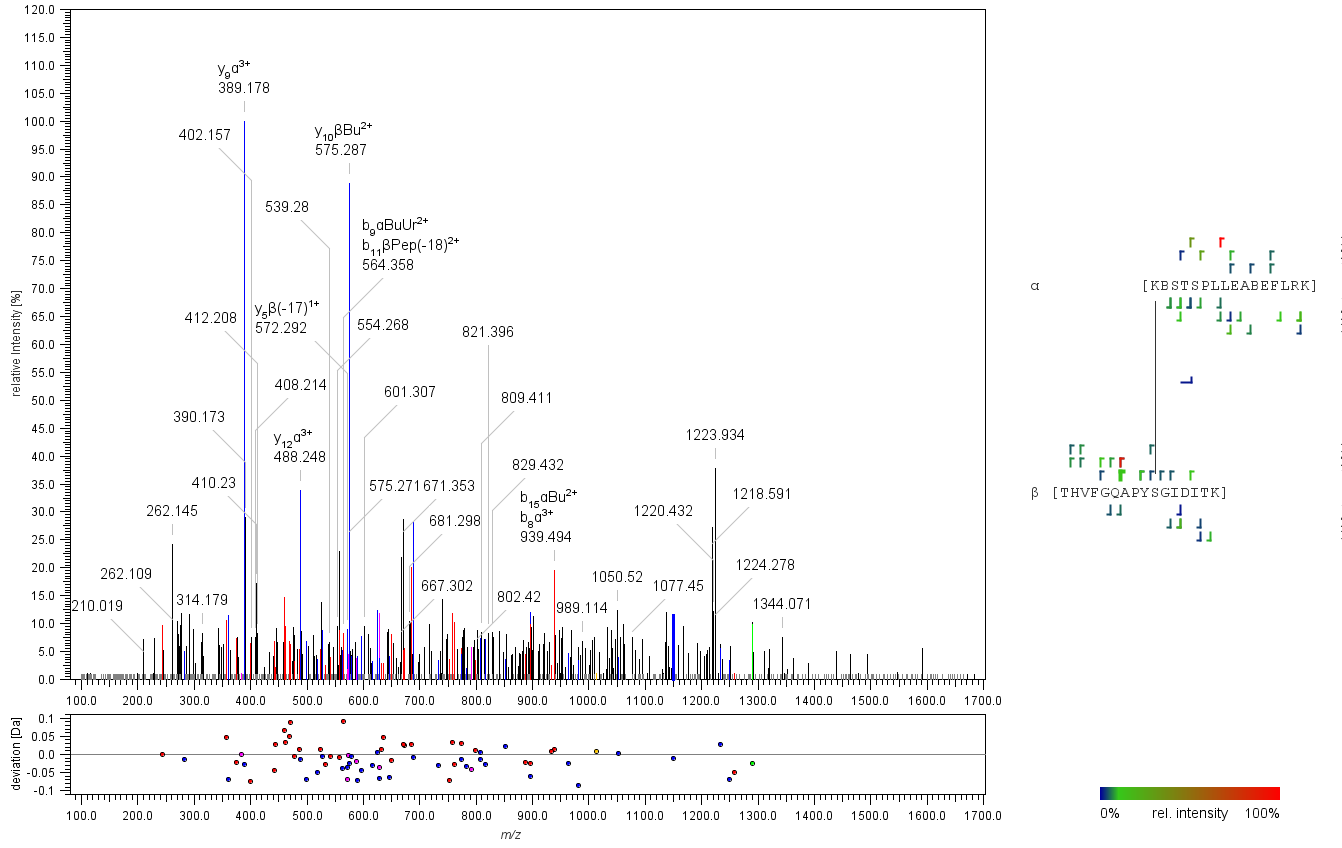

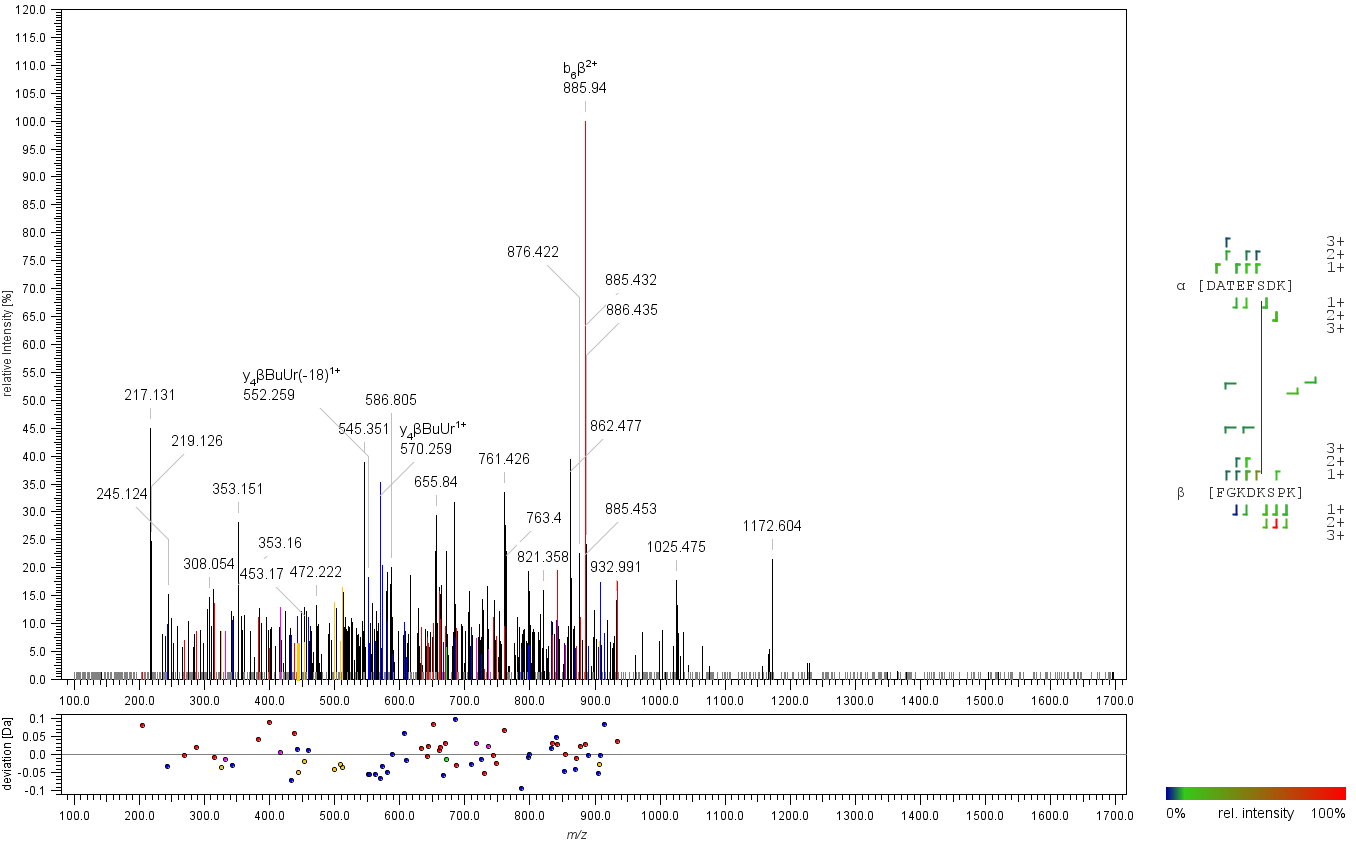


**Fig. S3.** Annotated tandem mass spectra of two intermolecular cross-linked peptides by MS-cleavable homobifunctional disuccinimidyl dibutyric urea (DBSU) reagent. Several peptide backbone and peptide-cross-linker (Bu and BuUr) fragments were matched using the MeroX software.


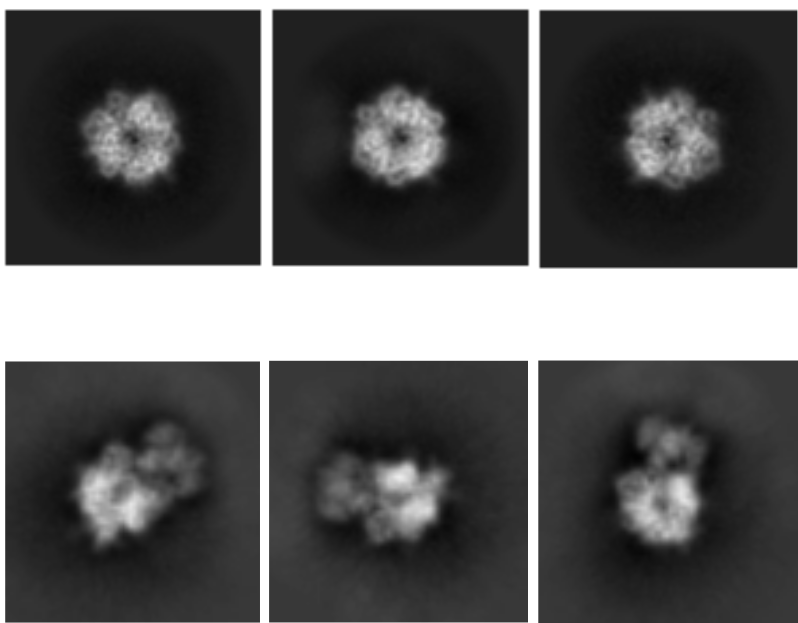


**Fig. S4.** 2D class averages showing hexon without no hLF bound (top), and bound to hLF (below). Conformational changes in hexon suggest an ‘opening-up’ of the hexon top upon binding of hLF.


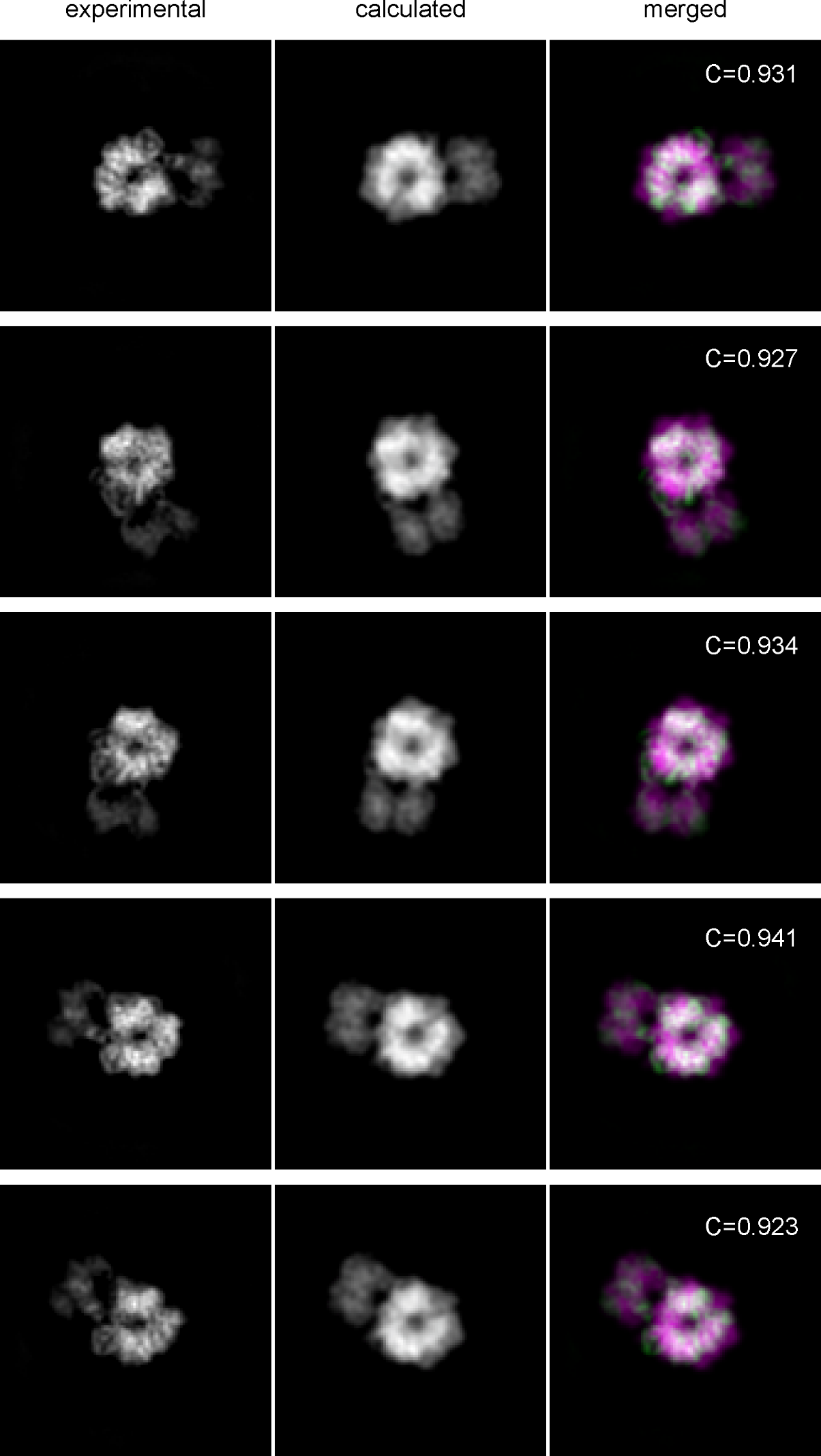


**Fig. S5.** Remaining lowest-energy solutions of hexon:hLF complex refined against 2D class averages of the complex. The left column shows the experimentally obtained 2D class averages for the hexon:hLF, the central column shows the calculated back projections for the hexon:hLF complex obtained after docking and energy minimization, and the right column shows the result of superposition of experimental (green) and calculated (magenta) projections. Cross-correlation (C) of experimental and calculated projections is shown for individual projections


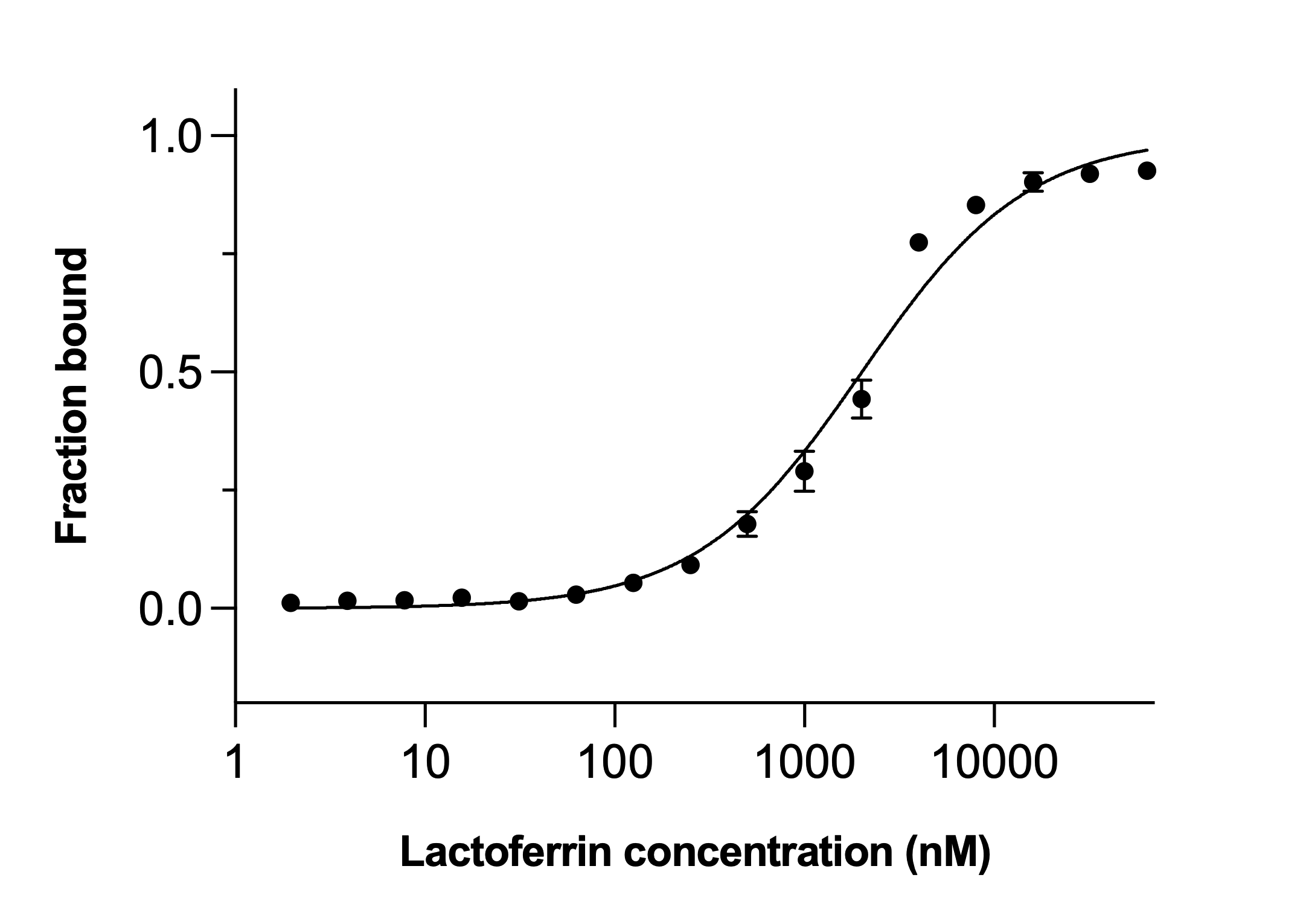


**Fig. S6.** Microscale thermophoresis measurement of the interaction between hexon and hLF at high ionic strength (buffer C + 0.02% Tween 20). Measurements were carried out as described in Materials and Methods. Due to lower affinity of the interaction at increased salt concentrations, the concentration range for hLF was adjusted accordingly. The LED power was set to 60%. The fraction of hLF bound to fluorescently labelled hexon was determined and plotted as dose response curve (n=3). The dissociation constant (KD) of the interaction was determined to be 1.9±0.3µM.


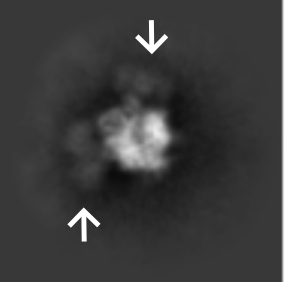


**Fig. S7.** A 2D class average showing 2 hLF copies (white arrows) bound to a hexon trimer.

hLF GRRRSVQWCAVSQPEATKCFQWQRNMRKVRGPPVSCIKRDSPIQCIQAIAENRADAVTLD 60

bLF APRKNVRWCTISQPEWFKCRRWQWRMKKLGAPSITCVRRAFALECIRAIAEKKADAVTLD 60

. *:.*:**::**** ** :** .*:*: .* ::*::* ::**:****::*******

hLF GGFIYEAGLAPYKLRPVAAEVYGTERQPRTHYYAVAVVKKGGSFQLNELQGLKSCHTGLR 120

bLF GGMVFEAGRDPYKLRPVAAEIYGTKESPQTHYYAVAVVKKGSNFQLDQLQGRKSCHTGLG 120

**:::*** **********:***:..*:************..***::*** *******

hLF RTAGWNVPIGTLRPFLNWTGPPEPIEAAVARFFSASCVPGADKGQFPNLCRLCAGTGENK 180

bLF RSAGWIIPMGILRPYLSWTESLEPLQGAVAKFFSASCVPCIDRQAYPNLCQLCKGEGENQ 180

*:*** :*:* ***:*.** **::.***:******** *: :****:** * ***:

hLF CAFSSQEPYFSYSGAFKCLRDGAGDVAFIRESTVFEDLSDEAERDEYELLCPDNTRKPVD 240

bLF CACSSREPYFGYSGAFKCLQDGAGDVAFVKETTVFENLPEKADRDQYELLCLNNSRAPVD 240

** **:****.********:********::*:****:* ::*:**:***** :*:* ***

hLF KFKDCHLARVPSHAVVARSVNGKEDAIWNLLRQAQEKFGKDKSPKFQLFGSPSGQKDLLF 300

bLF AFKECHLAQVPSHAVVARSVDGKEDLIWKLLSKAQEKFGKNKSRSFQLFGSPPGQRDLLF 300

**:****:***********:**** **:** :*******:** .******* **:****

hLF KDSAIGFSRVPPRIDSGLYLGSGYFTAIQNLRKSEEEVAARRARVVWCAVGEQELRKCNQ 360

bLF KDSALGFLRIPSKVDSALYLGSRYLTTLKNLRETAEEVKARYTRVVWCAVGPEEQKKCQQ 360

****:** *:* ::**.***** *:*:::***:: *** ** :******** :* :**:*

hLF WSGLSEGSVTCSSASTTEDCIALVLKGEADAMSLDGGYVYTAGKCGLVPVLAENYKSQQS 420

bLF WSQQSGQNVTCATASTTDDCIVLVLKGEADALNLDGGYIYTAGKCGLVPVLAENRKSSKH 420

** * .***::****:***.*********:.*****:*************** **.:

hLF SDPDPNCVDRPVEGYLAVAVVRRSDTSLTWNSVKGKKSCHTAVDRTAGWNIPMGLLFNQT 480

bLF --SSLDCVLRPTEGYLAVAVVKKANEGLTWNSLKDKKSCHTAVDRTAGWNIPMGLIVNQT 478

. :** **.*********:::: .*****:*.********************:.***

hLF GSCKFDEYFSQSCAPGSDPRSNLCALCIGDEQGENKCVPNSNERYYGYTGAFRCLAENAG 540

bLF GSCAFDEFFSQSCAPGADPKSRLCALCAGDDQGLDKCVPNSKEKYYGYTGAFRCLAEDVG 538

*** ***:********:**:*.***** **:** :******:*:*************:.*

hLF DVAFVKDVTVLQNTDGNNNEAWAKDLKLADFALLCLDGKRKPVTEARSCHLAMAPNHAVV 600

bLF DVAFVKNDTVWENTNGESTADWAKNLNREDFRLLCLDGTRKPVTEAQSCHLAVAPNHAVV 598

******: ** :**:*:.. ***:*: ** ******.*******:*****:*******

hLF SRMDKVERLKQVLLHQQAKFGRNGSDCPDKFCLFQSETKNLLFNDNTECLARLHGKTTYE 660

bLF SRSDRAAHVKQVLLHQQALFGKNGKNCPDKFCLFKSETKNLLFNDNTECLAKLGGRPTYE 658

** *:. ::********* **:**.:********:****************:* *: ***

hLF KYLGPQYVAGITNLKKCSTSPLLEACEFLRK 691

bLF EYLGTEYVTAIANLKKCSTSPLLEACAFLTR 689

:*** :**:.*:************** ** :

**Fig. S8.** Sequence alignment of mature human (hLF) and bovine (bLF) lactoferrin. The alignment was prepared using Clustal O (1.2.4). * – identical, : – highly similar, . – similar
